# Impact of COVID-19 on maternal and neonatal outcomes: a population-based cohort study in Latvia

**DOI:** 10.1093/eurpub/ckac130.241

**Published:** 2022-10-25

**Authors:** I Zile-Velika, I Gavare, V Veisa, D Rezeberga

**Affiliations:** 1 Centre for Disease Prevention and Control, Riga, Latvia; 2 Riga Stradins University, Riga, Latvia; 3 The Riga Maternity Hospital, Riga, Latvia; 4 Riga East University Hospital, Riga, Latvia

## Abstract

**Introduction:**

Coronavirus disease (COVID-19) has affected many pregnant women worldwide. Pregnant women with COVID-19 infection belong to a vulnerable group with concerns about the effect of the disease on maternal and neonatal health.

**Objectives:**

To assess association of COVID-19 on maternal and neonatal outcomes in Latvia.

**Methods:**

Data source was Medical Birth Register. A total of 17206 birth data for 2021 were included in the data analysis.

**Results:**

2.1% (n = 358) women with COVID-19 (U07.1; U07.2) during pregnancy or delivery. COVID-19 infection was related to the following conditions during pregnancy - gestation diabetes (10.6% to 4.6%; p < 0.001); placental abruption (1.7% to 0.6%; p < 0.05). Birth outcomes for COVID-19 infected women showed that infection has led to the increased caesarean section (27.7% to 22.0%; p < 0.01), preterm birth (12.8% to 5.3%; p < 0.001); low birth weight ≤2499g rate (10.1% to 3.9%; p < 0.001); stillbirths (2.8% to 0.4%; p < 0.001) and newborn infections specific to the perinatal period (P35-P39) (9.5% to 5.2%; p < 0.05). Increased BMI (8.9% to 6.8%), fetal distress (3.9% to 2.4%); preeclampsia, eclampsia (3.2% to 2.1%), hypertension (2.8% to 2.0%), gestational hypertension (5.0% to 3.8%) more frequently were observed among COVID-19 infected patients but the difference was not statistically significant. COVID-19 associated with higher odds (adjusted by mother age, multiple births, gestational age, mode of delivery) of gestational diabetes (ORadj 2.3; 95%CI 1.7-3.4; p < 0.001), newborn infections specific to the perinatal period (ORadj 1.7; 95%CI 1.2-2.4; p < 0.01) and stillbirth (ORadj 4.0; 95%CI 1.9-8.2; p < 0.001).

**Conclusions:**

COVID- 19 during pregnancy is associated with higher risk of adverse maternal and perinatal outcomes. The study results of short-term pregnancy outcomes show importance of implementation of all recommended COVID-19 prophylactic measures by public health specialists and clinicians during antenatal care.

**Key messages:**

- COVID- 19 during pregnancy is associated with higher risk of adverse maternal and perinatal outcomes.
- Study results of short-term pregnancy outcomes show importance of implementation of all recommended COVID-19 prophylactic measures by public health specialists and clinicians during antenatal care.

